# On Acute Nephritis in Children

**Published:** 1916-05-27

**Authors:** 


### ON ACUTE NEPHRITIS IN CHILDREN.

### The Importance of Urinalysis in Diseases of the Young.

There is one form of acute kidney disease in children which is familiar to every practitioner. It is seen perhaps most frequently during convalescence from scarlet fever. Œdema, scantiness of urine, albuminuria, and often hæmaturia form its most prominent characteristics, and have a meaning which is quite beyond dispute. Some will call the condition acute nephritis, some will name it acute Bright's disease, but all will agree on the essential diagnosis and on the principles which must govern treatment. On the practical side it may here be recalled that the value of leeches, if used early, is a very considerable one. Eight to a dozen of them over the loins will often clear up the symptoms with surprising rapidity, and it is astonishing how little support this measure finds in many text-books.

Scarlet fever, however, is not the only poison responsible for an acute nephritis. Other infectious diseases must take their place in the same evil category, and it is not necessary to enumerate them. Whether diphtheria must be included in the group is perhaps uncertain, but the disease is one in which albuminuria is common, even at a very early stage, and the point is one of diagnostic importance. Pneumonia, too, has often albuminuria among its consequences, and here occasionally there is definite evidence of nephritis in the shape of œdema and tube-casts. Another disease which may be quickly followed by nephritis is tonsillitis, and this is the more important as tonsillitis is usually grouped among the minor maladies. There is one clinical rule which ought never to be neglected. It is that in every case of tonsillitis a careful watch should be kept both on the kidneys and the heart. Whether tonsillitis is always "rheumatic" may be a matter for academic discussion, but it is certainly in early life sometimes the first event in a programme which may include polyarthritis and endocarditis. And similarly now and again it may be followed by clear evidence of nephritis. Hence the justification for the rule just stated. Cold and damp and chill have a classical position as causes of acute inflammation of the kidneys, though modern pathology is apt to jeer at the suggestion.

The general picture above sketched has little chance of misinterpretation. But there are certain departures from it which if not remembered may lead to error. One of the most startling of these is found in the proposition that nephritis may exist without albuminuria. That albuminuria does not always mean nephritis is, of course, a commonplace of medical experience, but the converse truth is not so generally known. Yet it stands on secure evidence. Part of this evidence is provided by *post-mortem* examinations in which manifest nephritis was discovered though the patient during life had been free from albuminuria. There are, further, well-authenticated records in which not only tube-casts in pathological numbers have been present, but also considerable œdema or anasarca. There exists a more or less vague notion that the dropsies of Bright's disease are due to inefficient elimination of fluid through the kidneys, but in all probability this view is quite incorrect. Far more probable is the suggestion that both the renal and the skin conditions are dependent on some poison or poisons, and that the incidence of these varies in different cases. If this is accepted, there is no great difficulty in allowing that while generally prejudice is inflicted in both of these directions, it may well be that in an individual instance the stress will fall mainly on the cutaneous vessels, and to a slight degree only on the kidneys. In such circumstances anasarca would be the conspicuous clinical fact, with nothing but tube-casts or renal cells to indicate renal disturbance. Whatever be the explanation, the facts are beyond question, and they show that even with such a strong suggestion of acute nephritis as general anasarca, the urine may be free from albumin, and that direct evidence of nephritis may be limited to the presence of tube-casts and renal cells. Hence, the practitioner must be prepared for a diagnosis of nephritis in spite of the absence of albuminuria.

### Another Diagnostic Pitfall.

The other side from which the risk of error comes is the possibility of acute nephritis without œdema. When the latter exists even the tyro is on the alert, for the association of œdema and albuminuria is one of the earliest letters in the professional alphabet. Further, the occurrence of chronic nephritis without dropsy is a familiar event. Chronic nephritis, however, is not common in children. Hence there has grown up a kind of rough presumption that if the child is free from œdema it cannot be suffering from nephritis. Certainly such a presumption is quite without foundation, and it would soon disappear were the examination of the urine in young patients made as much a matter of routine practice as is such examination in adults. The position must be stated in still more emphatic terms. Not only may œdema be absent with an attack of acute nephritis, but there may be also an entire absence of symptoms suggesting kidney disease, and hence unless the urine is examined the true nature of the case may be entirely overlooked. Gastro-intestinal disturbances are perhaps the most usual and most prominent events in these cases, and there may be fever extending over several days. Unless there is hæmaturia, and this in sufficient degree to attract the attention of the mother or nurse, there is little likely to be any symptom proposing renal disease, for quantitative alteration of the urine in children, unless extreme, is generally overlooked. In occasional instances the child's general condition is one of general seediness rather than of serious illness, and here the opportunity for error is still more conspicuous. There follows the general moral that in a scheme of clinical examination applied to children systematic testing of the urine ought to find a regular place.

